# The Quantified Behavioral Test—A Confirmatory Test in the Diagnostic Process of Adult ADHD?

**DOI:** 10.3389/fpsyt.2020.00216

**Published:** 2020-03-20

**Authors:** Nathalie Brunkhorst-Kanaan, Moritz Verdenhalven, Sarah Kittel-Schneider, Isabella Vainieri, Andreas Reif, Oliver Grimm

**Affiliations:** ^1^Department of Psychiatry, Psychosomatic Medicine and Psychotherapy, University Hospital, Goethe University, Frankfurt, Germany; ^2^Department of Psychiatry, Psychotherapy and Psychosomatic Medicine, University Hospital, University of Würzburg, Würzburg, Germany; ^3^Social, Genetic and Developmental Psychiatry Centre, Institute of Psychiatry, Psychology and Neuroscience, King’s College London, London, United Kingdom

**Keywords:** ADHD, ADHD differential diagnosis, ex-Gaussian analysis, continuous performance test, QbTest^®^, naturalistic sample

## Abstract

The differential diagnosis of attention deficit hyperactivity disorder (ADHD) in adulthood is complicated by comorbid disorders, but also by the overlapping of main symptoms such as inattentiveness, impulsivity, and hyperactivity with other disorders. Neuropsychological tests like continuous performance tests (CPT) try to solve this dilemma by objectively measurable parameters. We investigated in a cohort of n=114 patients presenting to an ADHD outpatient clinic how well a commercially available CPT test (QbTest^®^) can differentiate between patients with ADHD (n=94) and patients with a disconfirmed ADHD diagnosis (n=20). Both groups showed numerous comorbidities, predominantly depression (27.2% in the ADHD group vs. 45% in the non-ADHD group) and substance-use disorders (18.1% vs. 10%, respectively). Patients with ADHD showed significant higher activity (2.07 ± 1.23) than patients without ADHD (1.34 ± 1.27, dF=112; p=0.019), whereas for the other core parameters, inattention and impulsivity no differences could be found. Reaction time variability has been discussed as a typical marker for inattention in ADHD. Therefore, we investigated how well ex-Gaussian analysis of response time can differentiate between ADHD and other patients, showing, that it does not help to identify patients with ADHD. Even though patients with ADHD showed significantly higher activity, this parameter differed only poorly between patients (accuracy AUC 65% of an ROC-Curve). We conclude that CPTs do not help to identify patients with ADHD in a specialized outpatient clinic. The usability of this test for differentiating between ADHD and other psychiatric disorders is poor and a sophisticated analysis of reaction time did not decisively increase the test accuracy.

## Introduction

Attention deficit hyperactivity disorder (ADHD) is the most frequently diagnosed childhood neurodevelopmental disorder defined by the core symptoms inattention, hyperactivity, and impulsivity leading to negative consequences for life and health in patients (1). Symptoms often continue into adult life where they contribute to severe distress among patients like higher school drop-out rate, higher unemployment rate (2, 3), divorces, accidents (4, 5), and a more frequent development of other psychiatric diseases (5, 6). Recent studies confirm that symptoms persist in 30%–40% of adult cases (1, 7, 8) leading to a prevalence of 3%–4% in the general population (9).

Due to the heterogeneity of symptoms and the high comorbidity with other psychiatric diseases like anxiety disorder, depression, bipolar disorder, borderline personality disorder (BPD), and substance use disorder (SUD) (10, 11), the diagnosis of adult ADHD patients can be challenging. Furthermore, core symptoms of ADHD can be also characteristic for other psychiatric disorders (12). For instance, cognitive deficits like inattention, forgetfulness, low attention span can also be found in patients who suffer from schizophrenia, bipolar disorder, or depression (13, 14). Similarly, impulsive behavior and emotional lability are also characteristic symptoms shared between ADHD and BPD (15). Diagnosis of ADHD is based on assessment of symptoms and impairment in childhood (16–18). As recommended by international guidelines, screening interviews, self-report questionnaires, and diagnostic interviews are commonly used diagnostic tools for the diagnosis of ADHD (19, 20). However, these are compromised in varying degree by subjective memory and evaluation biases which can affect specificity and discriminative ability of these tests (21–24).

Thus, objective neuropsychological tests, like CPTs (continuous performance tests), to measure inattention and impulsivity were added to the diagnostic process. Even though CPTs have been frequently used as a diagnostic tool, their use has been criticized due to the lack of specificity and sensitivity for adult ADHD (25), especially when patients suffer from other psychiatric diseases. Other critics to the CPT have been related to the lack of psychometric measures specific for hyperactivity (16, 25–28) in contrast to pure inattention sensitive measures. While CPTs in its classic form are valid for inattentiveness, they lack specificity for hyperactivity which is an important core feature of ADHD and helps to discriminate ADHD from other comorbidities (15). Among measures of inattentiveness, high reaction time (RT) variability, which is a measure that reflects attentional lapses, was found to be increased in adults and children with ADHD using a wide range of tests including CPTs (29–31). The Quantified Behavioral Test (QbTest^®^, marketed by Qbtech) is a further developed CPT which is frequently used in clinical practice. Measures of motor activity were added by recording movements of a subject from a reflective marker attached to a headband while performing the CPT tasks (32). Head movement and CPT data are then analyzed *via* a principal component analysis and a consecutive distribution into a normal distribution. These parameters (Qb-activity, Qb-impulsivity, and Qb-inattention) are validated in a normal population, so that test results above the 93^rd^ percentile are suggestive of ADHD (28). There is evidence for the validity of the QbTest^®^ to identify patients with ADHD in adults and children when compared to healthy controls (33). But, in comparison to clinical patients, the QbTest^®^ showed a poor discriminative ability (15, 29, 32).

Computationally sophisticated analysis of CPT RTs might provide additional information in discriminating among different disorders. Previous studies showed RT variability (RTV) to be consistently increased in people with ADHD and other disorders with attention problems like autism or bipolar disorder (32, 34). The RT distribution can be further decomposed using the ex-Gaussian approach. This applies the convolution of a normal and an additional exponential function to the RT data. As a result, the ex-Gaussian decomposes the RT distribution into three parameters: Mu (µ) representing the mean of the normal component (=average performance), Sigma (σ) which represents the variability of the normal distribution, and Tau (τ) corresponding to the variability of the exponential function. Conventionally, µ and σ represent the variability of the most frequent responses, whereas τ reflects the variability of the infrequent long responses. Analyses on RT data from participants with ADHD suggest that ultra-long RTs (τ) are specific for ADHD (35, 36).

Thus, the aims of our investigation were to (1) examine the ecological validity of the QbTest^®^ which means to what extent the results of the QbTest^®^ can be generalized to real-life patients that undergo diagnostic assessment for adult ADHD in an outpatient clinic. This we test by looking at the receiver-operating curves (accuracy) and subsequent sensitivity and specificity. We were especially interested which of the three components (hyperactivity, inattentiveness, or impulsivity) are the most accurate in diagnosing ADHD. And (2), in addition to standard-analysis data, we wanted to explore whether a more sophisticated data analysis like fitting an ex-Gaussian distribution model to our RT could provide better accuracy.

## Methods

### Participants

Data from structured diagnostic interview for ADHD in adults (DIVA 2.0), Wender-Utah-rating-scale short form (WURS-K) (37), adult ADHD self-report scale (ASRS) (9), and the QbTest^®^ were collected at the Department of Psychiatry, Psychosomatic Medicine, and Psychotherapy from a naturalistic sample of 114 patients (**Table 1**) undergoing diagnostic assessment for adult ADHD between July 2017 and July 2018. Participants were referred by psychiatrists, general practitioners, or psychotherapists for diagnostic evaluation to our specialized outpatient clinic.

**Table 1:** Patient characteristics.

	ADHD	Non-ADHD	p-Value
N (%)	94 (82.5%)	20 (17.5%)	
Sex (% female)	42.6	60.0	0.155
Age (M(years) ± SD)	34.7 ± 11.05	35.8 ± 10.6	0.69
Depression (n (%))	26 (27.7)	9 (45)	0.127
SUD (n (%))	17 (18.1)	2 (10)	0.466
Bipolar (n (%))	2 (2.1)	1 (5)	0.776
Other (n (%))	8(8.5)	3 (15)	0.155
Healthy (n(%))	**0(0)**	**8(40)**	**<0.001**
QbIna (M ± SD)	1.21 ± 1.17	1.00 ± 1.26	0.369
QbImp (M ± SD)	0.63 ± 1.12	0.35 ± 0.85	0.281
QbAct (M ± SD)	**2.07 ± 1.23**	**1.34 ± 1.27**	**0.019**
µ (M ± SD)	471.57 ± 140.94	463.31 ± 149.83	0.545
σ (M ± SD)	78.42 ± 49.64	67.59 ± 57.52	0.144
τ (M ± SD)	165.71 ± 52.84	159.53 ± 67.06	0.259

The ADHD group is composed of 94 (82.5%) patients who, in the end, met the criteria for an ADHD diagnosis. Among these, 49 (52.1%) patients had no other psychiatric disorder while 45 (47.9%) of the ADHD cases displayed a comorbidity with at least one other psychiatric disorder, five of them (4.4%) had at least two other psychiatric disorders. 26 (22.8%) had ADHD and Depression, 17 (14.9%) ADHD, and SUD, two (1.8%) ADHD and bipolar disorder (BD), eight (7.0%) ADHD and other disorders such as posttraumatic stress disorder, somatization disorders, or obsessive-compulsive disorder. The patients with affective comorbidities all suffered from moderate to severe depressive episodes at the time of examination.

The “Non-ADHD-Group,” where adult ADHD was ruled out during the diagnostic process, consists of 20 patients (17.5%). Eight patients (7%) had no psychiatric disorder according to ICD-10 criteria, nine (7.9%) patients suffered from depression, two (1.8%) patients from a SUD, one patient met the criteria for a bipolar disorder (0.9%) and three (2.6%) patients suffered from other psychiatric disorders.

Informed written consent was obtained from all subjects. The study followed an observational parallel group design. The study was approved by the Ethics Committee of the University Hospital Frankfurt (No. 425/14).

### Diagnostic Assessment

The diagnostic process consisted of an open clinical interview by an experienced clinician to obtain a broad view of the medical history concerning current and former symptoms and associated impairment and functioning, history of substance abuse, psychiatric comorbidities, somatic disorders, and family history of ADHD. To ascertain symptoms in childhood all patients were asked to hand in primary school reports or childhood medical documents.

For the main diagnostic assessment, the diagnostic interview for adult ADHD (DIVA 2.0) (38) was used. The DIVA is a semistructured interview developed for adults consisting of two parts: At first, current and childhood ADHD core symptoms are assessed (38). Then, the impairment due to ADHD symptoms in five domains of functioning is evaluated (school/work, social contacts, free-time/hobbies, self-confidence). If five or more criteria are met for either inattention and/or hyperactivity/impulsivity and impairment in at least two domains of functioning in both childhood and adulthood, a diagnosis of ADHD is plausible [using DSM-5 criteria (39)].

Additional information concerning symptoms of ADHD in childhood were obtained from the short form of the Wender Utah Rating Scale (WURS-K) questionnaire. Finally, all patients were tested by the QbTest^®^ (Qbtech, Stockholm, Sweden) on an additional appointment. A diagnosis of ADHD was established when the DIVA interview was positive, no other diagnoses explained the symptomatology better, and when clinical judgement was in line with the DIVA.

### QbTest^®^

The QbTest^®^ is a variant of a CPT. It is based on a one-back task. Apart from the classical assessment of commission and omission errors, the QbTest^®^ additionally measures motor activity with an infrared camera and a reflector attached to a headband during the 20 min of testing. The QbTest^®^ presents raw scores from measures of movement (time active, distance area, microevents, and motion simplicity), raw scores of inattention (omission errors, RT, and RT variation during the second half of the test) and impulsivity raw scores (commission errors, normalized commission errors, and anticipatory responses). From those raw scores the three cardinal parameters Qb-activity (QbAct), Qb-inattention (QbIna), and Qb-impulsivity (QbImp) are derived by performing a principal component analysis. These parameters are transformed into normally distributed Q-scores implicating information about the difference between the raw score of a patient compared to scores of a gender- and age-controlled group (40). The 93rd percentile, which equals a Qb-Score of 1.5 is the recommended cut-off threshold in differentiating between a healthy and an ADHD population (15). There are no recommended thresholds for specifying ADHD in contrast to other psychiatric disorders.

### Exponential-Gaussian-Analysis

The “ex-Gaussian” RT distribution model, a convolution of both components of a Gaussian distribution and an exponential distribution, is proposed to better define RT data. The three ex-Gaussian parameters include the following: μ (mu) and σ (sigma), mean and SD of the Gaussian portion, respectively; and τ (tau), mean of the exponential portion. The value of τ captures the tail of the RT skewed distribution and provides better measures of increased RTV in terms of extremely slow but infrequent responses, while µ represents the mean of the normal distributed RT and σ represents the variability of responses both above and below the mean, that is, normal distributions. Larger τ consistently and stably differentiates ADHD from controls across experimental tasks.

## Statistical Analysis

Data were analyzed with SPSS version 22 (IBM Corp. Released 2013, IBM SPSS Statistics for Windows, Version 22.0. Armonk, NY). To test for normal distribution of the cardinal Qb parameters the Kolmogorov-Smirnov test was used. The t-test and the Mann-Whitney U-test were used for group comparison. For further analysis a multiple logistic regression analysis of independent variables was carried out. The variables of the ex-Gaussian analyses were extracted using a maximum-likelihood algorithm and implemented using the quantile maximum probability estimator (QMPE) software, an open source ANSI Fortran program for response time distribution estimation (41). For evaluation of the discriminative power of the QbTest^®^ and the ex-Gaussian parameters an ROC (receiver operating characteristic) curve and the area under the curve were computed. The clinical ADHD diagnosis was used as the external criterion for the calculation of sensitivity, specificity, and area under the ROC curve (AUC) for activity, impulsivity and inattention and µ, σ, and τ.

## Results

### Sample Characteristics

There was a higher proportion of female patients in the non-ADHD-group, not reaching statistical significance in a Chi-square test (X^2^(1)=2.023 p=0.155). Furthermore, a higher rate of depression was found in the non-ADHD group and a higher percentage of patients with SUD in the ADHD group (**Table 1**). However, there was no significant difference in a Chi-square test between the two groups concerning comorbidities (Depression (X^2^(1)=2.331 p=0.127), Bipolar (X^2^(1)=0.531 p=0.466), SUD (X^2^(1)=0.776 p=0.378), other (X^2^(1)=2.023 p=0.155). In the non-ADHD group were eight subjects without psychiatric disorders, reaching statistical significance in a Chi-square test (X^2^ (1)=40.438 p < 0.001) The age of the patients in the ADHD group (M=34.7 SD=11.05) did not differ from the age of the non-ADHD group (M=35.8 SD=10.6) in an independent-sample t-test (t(112)=0.399 p=0.69) (**Table 1**).

The core variables QbActivity (QbAct) and QbImpulsivity (QbImp) showed Gaussian distribution according to the Kolmogorov-Smirnoff test (QbAct df=114, p=0.175; QbImp df=114, p=0.200). QbInattention (QbIna) showed no normal distribution (QbIna df=114, p=0.046).

There were no statistically significant differences between patients with ADHD (0.63 ± 1.12) and patients without ADHD for QbImp (0.35 ± 0.85; dF=112; p=0.281) and QbIna (with ADHD 1.21 ± 1.17; without ADHD 1.00 ± 1.26; dF=112; p=0.369). Patients with ADHD showed significantly higher QbAct-values (2.07 ± 1.23) than patients without ADHD (1.34 ± 1.27, dF=112; p=0.019) (**Table 1**). The ex-Gaussian parameters showed no Gaussian-distribution in a Kolmogorov-Smirnov-Test (µ: p=0.001; σ: p=0.003; τ: p=0.001). In none of the ex-Gaussian parameters a statistically significant difference in a Mann-Whitney-U-Test between patients with ADHD and patients without ADHD (**Table 1**) could be found.

A multiple regression was carried out to investigate whether psychiatric disorders, sex or age represented as independent variables significantly influenced parameters measured by the QbTest^®^. While ADHD contributed significantly to QbActivity (β=0.711, p=0.013), all other independent variables did not (**Table 2**). Thus, an ADHD diagnosis leads to higher activity values measured in the QbTest^®^. In contrast, neither QbInattention nor QbImpulsivity were influenced by any independent variable. There was no significant correlation for ADHD and QbImpulsivity (β=0.129, p=0.648) as well as for ADHD and QbInattention (β=0.262, p=0.358);. In the regression model with QbImp as dependent variable, neither disease (p > 0.384, dF=124), nor age or gender (p > 0.335, df=124) reached significance, the same applies for QbIna: disease (p > 0239, df=124); age or gender (p > 0.099, dF=124). A detailed list of regression results is found in the **Supplemental Tables 1** and **2**. The same regression analysis was carried out for µ, σ, and τ. Detailed results are presented in the **Supplemental Tables 3**–**5**. In summary, there was no significant correlation of the independent variables with µ, σ, and τ.

**Table 2:** Coefficients and statistics of independent variables of the multiple logistic regression model for prediction of QbAct. n=114.

	Not standardized coefficients		95,0% confidence intervals for B		Standardized coefficients	T	Sig.
	Regression-coefficient B	Sth. Error	Lower bound	Upper bound	β		
(constant)	1.091	0.572	−0.040	2.223		1.909	0.059
ADHD	**0.711**	**0.283**	**0.150**	**1.272**	**0.221**	**2.510**	**0.013**
Depression	0.375	0.262	−0.143	0.893	0.139	1.434	0.154
SUD	−0.057	0.302	−0.656	0.541	−0.017	−0.190	0.850
Bipolar	−0.618	0.743	−2.088	0.853	−0.073	−0.831	0.407
Other	−0.265	0.376	−1.010	0.480	−0.065	−0.704	0.483
Gender	0.068	0.232	−0.392	0.528	0.027	0.294	0.769
Age	0.005	0.011	−0.016	0.026	0.041	0.456	.649

### Sensitivity and Specificity of the QbTest^®^ in Real-Life Patient Groups

**Figure 1:**
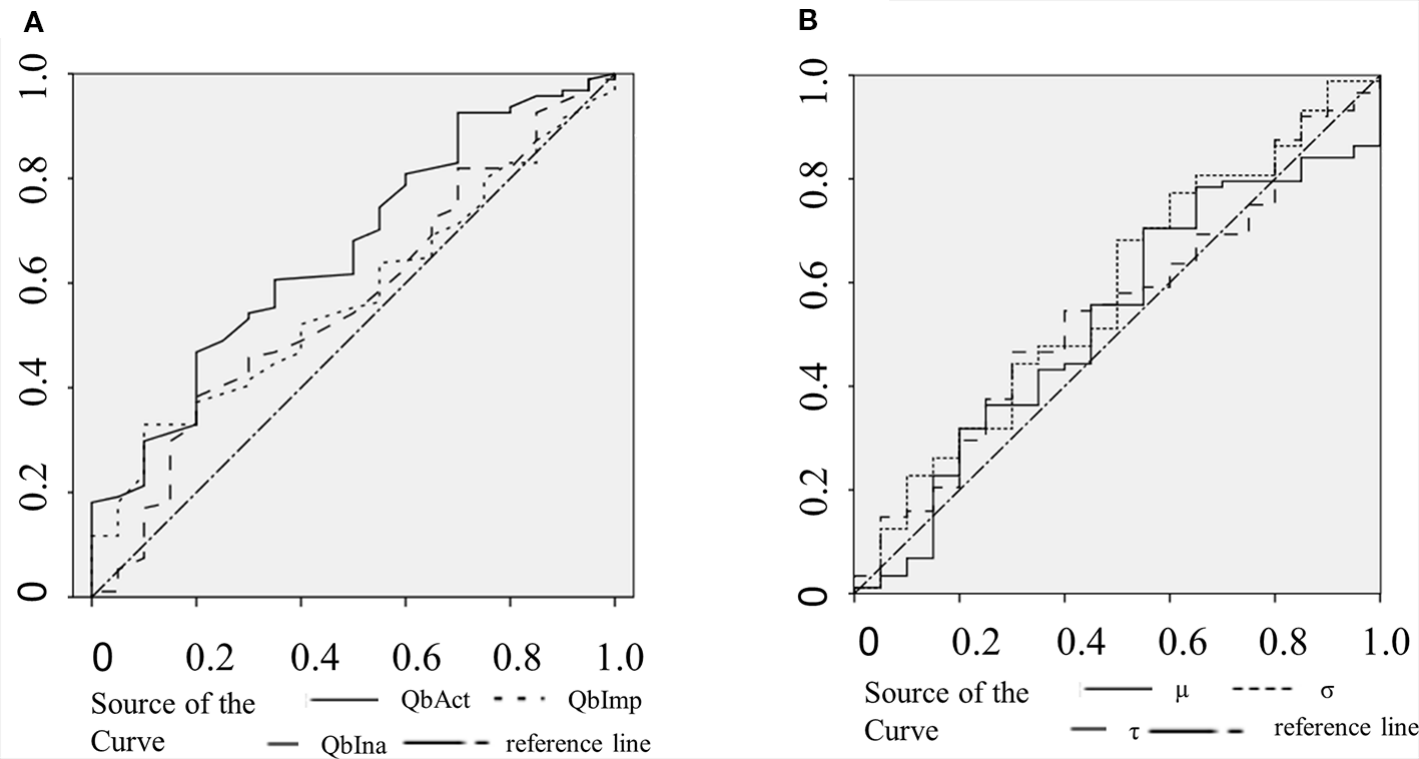
Receiver operating characteristic (ROC) curves. **(A)** ROC curves of QbAct (solid), QbImp (dotted), and QbIna (dashed). **(B)** ROC curves of µ (solid), σ (dotted), and τ (dashed).

**Figure 2:**
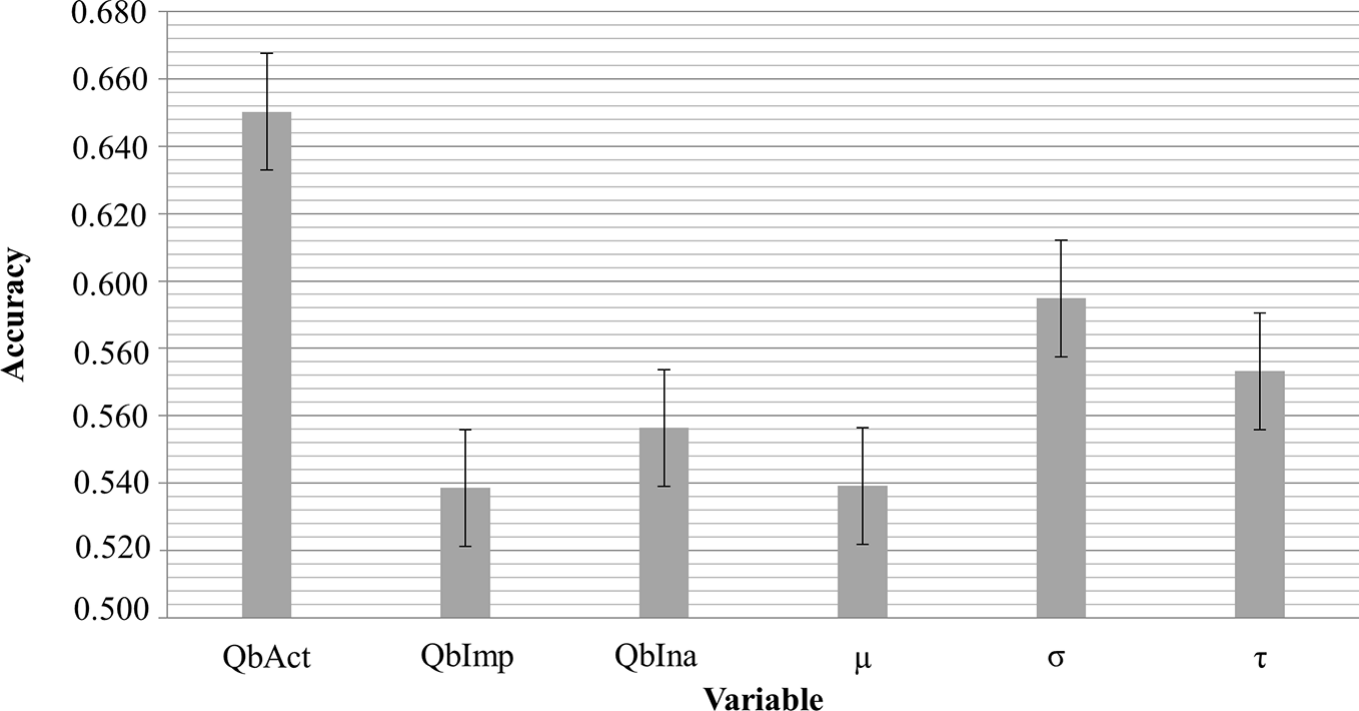
Comparison of the different measures acquired by the QbTest^®^.The bars show the 95% CI.

The clinical ADHD diagnosis was used as the external criterion for the calculation of sensitivity, specificity, and the receiver operating characteristic (ROC) curve for QbAct, QbImp, and QbIna. The only variable with prognostic value according to the ROC-curve seems to be QBAct, reaching a sensitivity of app. 50% at a specificity of app. 70% (**Figure 1A**). As QbImp and QbIna, the ex-Gaussian variables µ, σ, and τ cross or touch the reference line several times, indicating a similar prognostic value as chance (**Figure 1B**). To further quantify the prognostic values the AUCs were calculated, corresponding with the diagnostic accuracy (**Figure 2**). With an AUC of 0.65 ROC, QbAct shows the best discriminative power compared to the other QbTest^®^ items. It is distinct from chance (AUC=0.5), but still performs poor (42). Using a cut-off score of 1.5, as recommended by the developers, we found a sensitivity for QbAct of 68% and a specificity of 48%. The AUC of QbIna (0.556) and QbImp. (0.539) have poor discriminative power and are hardly better than chance (AUC=0.5).

In our clinical practice, we considered higher cut-off for QbAct useful: a cut-off of 2.35 gives a specificity of 76% (sensitivity 48%) and a cut-off of 2.95 gives a specificity of 92% (sensitivity 28%).

Analyzing the discriminative ability of RTV all three ROC-curves show an AUC smaller than 0.6 (σ:0.595; τ: 0.573; µ:0.539).

## Discussion

The aim of our study was to investigate the ecological validity of the QbTest^®^ in a naturalistic setting in an outpatient clinic. The lack of CPTs to differentiate between ADHD and other psychiatric disorders was already described by others (43, 44). In contrast to the study presented here, these studies were performed on children and not adults and with CPTs that do not measure hyperactivity. Furthermore none of the older studies used more sophisticated analyses of the RT as parameter for inattention as represented by the ex-Gaussian analyses. Our hypothesis was that the QbTest^®^, objectively measuring all three core parameters of ADHD, could serve as an objective neuropsychological test to discriminate between ADHD and non-ADHD patients in real-life adult patients with the typical heterogeneity of symptoms and comorbidities. This would be useful in the clinical setting; discrimination between healthy controls and ADHD patients is rather an epidemiological, but not a clinical problem, but differential diagnosis between different patient groups commonly is.

Our data show that QbActivity is the only parameter to significantly distinguish ADHD from other clinical patient groups. The regression analysis shows, that ADHD is the only psychiatric condition correlating positively with QbAct meaning that a diagnosis of ADHD contributes to a higher QbAct index.

In addition to the comparison of the different groups we were interested in the discriminative power of the QbTest^®^ to calculate the true positive and false positive rate, the latter reflecting non-ADHD probands (other psychiatric disorders, or no mental disorder at all) with positive QbTest^®^ result. In contrast to the use of the QbTest^®^ as a diagnostic tool, our data shows that it has a very low discriminative power. QbAct has an AUC of 0.65 (sensitivity 76%; specificity 40%), and the AUC of QbImp (0.539) and QbIna (0.556) are at chance level. Taken together, these values render this test not clinically useful at least in a secondary care setting.

A recent line of research indicates that technically more sophisticated modes of analysis like ex-Gaussian modelling are able to capture ADHD-specific variations in RT during cognitive demanding tasks (45, 46). However, none of these studies investigated whether ex-Gaussian parameters could help differentiate between ADHD and non-ADHD patients. Our analysis shows, that the ex-Gaussian parameters µ, σ, and τ are not useful in differentiating between patients with ADHD and patients without ADHD based on their ROC accuracy. Reflecting the standard deviation of the Gaussian distribution (µ and σ) and of the exponential distribution (τ), they indeed seem to capture ADHD-specific features. However, these features were even less specific than the discriminatory power of QbAct. As RTV shows a U-shape across ages, it might be more useful in children and elderly (47).

Previous studies investigating the clinical usefulness of the QbTest^®^ showed varying results, depending on whether ADHD patients were compared to healthy controls or to clinical controls. Edebol et al. reported a sensitivity of 86% and a specificity of 83% for the QbTest^®^ when comparing ADHD patients to healthy controls, which decreased to a specificity of 41% when comparing ADHD patients to patients with a bipolar II disorder and to a specificity of 36% when comparing ADHD patients to disconfirmed ADHD patients (15, 28). Other studies including clinical controls showed a poor general ability (29, 32) regarding the discriminative validity of the QbTest^®^ due to several reasons; most of them were explained by the effect of comorbidities (29, 32) and other neurodevelopmental disorders (48). Our data are in line with these findings.

With respect to the discriminative validity of each core parameter, our results are similar to previous studies in which QbAct was described as the objective measure of ADHD (29, 33) with the best ability to distinguish between ADHD patients versus clinical controls (15). Despite of these results from QbTest^®^ studies, further support of the importance of hyperactivity was described in a meta-analysis of Murillo et al. on the potential value of objective locomotor measures as “biomarker” for ADHD (30). The meta-analyses showed significantly increased head movement in children and adolescents with ADHD, as well as in adults with ADHD compared to “normally” developed controls. In an additionally conducted case-control study, a correlation between locomotor activity and errors and accuracy in a go/no-go task could be shown (30). This correlation was confirmed by an fMRI study in which a significant correlation between head motion and maternal-reported as well as self-reported Inattention, Hyperactivity and Impulsivity could be shown (49). The authors suggest that altered head motion could represent a valuable endophenotype and a risk/trait biomarker of hyperactivity, impulsivity and inattention in ADHD or other neurodevelopmental disorders in RS-fMRI studies (49, 50).

Measures of impulsivity and inattention as given by the CPT standard cardinal parameters or by more sophisticated ex-Gaussian analysis are not very successful in differentiating between patients with ADHD and other psychiatric disorders. This raises the question whether these constructs are really overlapping with clinical symptoms of inattention and impulsivity.

Taken together, these studies show that activity is the most objective and hence best measurable symptom in ADHD patients and occurs constantly in all ages. On the other hand, some studies emphasize that hyperactivity is not an ADHD specific marker, but rather an indicator for further assessment of coexisting psychiatric diseases. In our study, while the non-ADHD group was close to the cut-off score for QbAct, only the ADHD-sample reached high hyperactivity scores. A very high specificity of 92% can only be reached by lowering sensitivity to 28% (cut-off 2.95). Therefore, we suggest that patients above much higher thresholds than previously proposed (1.5) are correctly identified as ADHD cases in a naturalistic setting.

In our patient sample, both groups (ADHD and non-ADHD) included patients with psychiatric comorbidities and some patients had more than one comorbidity which however reflects clinical reality; our patient sample was representative of other clinical German ADHD population with respect to the prevalence of comorbid conditions (51, 52). The non-ADHD group almost reached the cut-off score for QbAct in our study. It might be possible that the results of our sample are influenced by different prevalence of comorbidities in each sample explaining the low sensitivity and specificity. Evidence for this assumption is provided in the study of Söderström et al. where the clinical control group performed worse than healthy control groups in the QbTest^®^ (32). Given the heterogeneity of clinical samples, larger sample sizes than n=114 are necessary to statistically clarify the impact of the comorbidities on the results of the QbTest^®^. Further studies with larger patient samples should investigate the effect of distinct comorbidities on neuropsychological testing.

While our sample was unevenly distributed between ADHD patients and non-ADHD patients, it should be kept in mind that we wanted to compare a highly selected patient population. All patients seeking ADHD treatment or assessment of ADHD diagnosis are at least partially transferred by other clinicians/psychiatrists when they display ADHD relevant symptoms. In this biased setting, tests which discriminate well between patients and healthy controls would fare worse. In our study, the QbTest^®^ was for inattention and impulsivity values close to a random ROC curve. This renders the test clinically useless. The hyperactivity score reached a somewhat better AUC accuracy of 65%. This must be compared to other clinical screening tests, for example the PSA-screening for prostate cancer, which has a very high sensitivity (95%) but a very low specificity (20%–30%). The QbTest^®^'s performance reminds on the test of brain-natriuretic-peptide (BNP) in the diagnosis of heart failure in an emergence setting. While the specificity is high, the accuracy is about 80%. As a consequence of this, clinicians do not base their diagnosis on BNP (53). Future studies should evaluate the QbTest^®^ against other standardized diagnostic instruments like DIVA-interview or WURS-k.

It has been critically discussed, whether neuropsychological testing under these circumstances makes sense at all in ADHD. A neuropsychological assessment to assess a behaviorally defined ADHD construct will always result in suboptimal accuracy of the instruments in question because of the heterogeneity in the functional level of patients behaviorally classified as having ADHD (54, 55).

In conclusion, our study shows that the QbTest^®^ is not able to discriminate between ADHD patients and non-ADHD patients in an outpatient clinic. As the QbTest^®^ currently is used in clinical practice especially under private reimbursement schemes, its application and clinical usefulness has to be critically discussed at present. Since the test is able to quantify the three core symptoms related to ADHD, it is valuable for research settings, even though a correlation between the measures of the QbTest^®^ and the impairment in daily life remains to be elucidated.

## Data Availability Statement

All datasets generated for this study are included in the article/**Supplementary Material**.

## Ethics Statement

The studies involving human participants were reviewed and approved by Ethics Committee of the medical Faculty University Hospital Frankfurt Goethe University. The patients/participants provided their written informed consent to participate in this study.

## Author Contributions

NB-K and MV wrote the manuscript, calculated statistical analyses of the QbTest^®^ parameters and the patient sample, and recruited and tested the patients. AR, SK-S, MV, and GO contributed design and conception of the study. GO coordinated and supervised the study, calculated statistical analyses of the QbTest^®^ parameters and the patient sample, recruited and tested patients, and drafted the manuscript. IV calculated the statistical analyses for the ex-Gaussian parameters and drafted the manuscript. SK-S and AR drafted the manuscript.

## Funding

Financial support for this study was received from the European Union's Horizon 2020 research and innovation programme under grant agreement 667302 (Comorbid Conditions of ADHD).The QbTest®was partially funded in the framework of the PRADA study which was funded by Medice Arzneimittel Pütter GmbH.

## Conflict of Interest

AR has received a research grant from Medice and served on advisory board and/or speaker's bureau from Medice, Shire/Takeda, Janssen, Servier, and neuraxpharm. SK-S has served on advisory board and/or speaker's bureau for Medice Arzneimittel Pütter GmbH and Shire/Takeda. GO has served on advisory board and/or speaker's bureau for Medice Arzneimittel Pütter GmbH.

The remaining authors declare that the research was conducted in the absence of any commercial or financial relationships that could be construed as a potential conflict of interest.
