# Supplementary material for: The Quantified Behavioral Test—A Confirmatory Test in the Diagnostic Process of Adult ADHD?
Source: Front Psychiatry. 2020 Mar 20;11:216. doi: 10.3389/fpsyt.2020.00216 (PMC7100366; doi:10.3389/fpsyt.2020.00216)
Supplement: Supplementary file 1 [file DataSheet_1.docx]

Supplement Table 1: Coefficients and statistics of independent variables of the multiple logistic regression model for prediction of QbImp. n=114.

|  | | not standardised coefficients | | 95% Confidence intervalls of B | | Standardised coefficients | T | Sig. |
| --- | --- | --- | --- | --- | --- | --- | --- | --- |
|  |  | Regression-coefficient B | Std.-Error | Lower bound | Upper bound | β |  |  |
|  | (constant) | .103 | .572 | -0.943 | 1.262 |  | .180 | .858 |
|  | ADHD | .129 | .281 | -0.438 | 0.655 | .043 | .458 | .648 |
|  | Depression | -.230 | .264 | -0.700 | 0.309 | -.090 | -.874 | .384 |
|  | SUD | .073 | .306 | -0.517 | 0.650 | .023 | .240 | .811 |
|  | Bipolar | -.304 | .734 | -1.712 | 1.154 | -.039 | -.415 | .679 |
|  | Other | -.015 | .372 | -0.725 | 0.727 | -.004 | -.039 | .969 |
|  | Gender | .228 | .236 | -0.267 | 0.629 | .094 | .968 | .335 |
|  | AgeD | .006 | .011 | -0.015 | 0.026 | .050 | .528 | .598 |

*Supplement Table 2*: Coefficients and statistics of independent variables of the multiple logistic regression model for prediction of QbIna. n=114.

|  | | not standardised coefficients | | 95% Confidence intervalls of B | | Standardised coefficients | T | Sig. |
| --- | --- | --- | --- | --- | --- | --- | --- | --- |
|  |  | Regression-coefficient B | Std.-Error | Lower bound | Upper bound | β |  |  |
|  | (constant) | .084 | .577 | -1.067 | 1.150 |  | .146 | .884 |
|  | ADHD | .262 | .283 | -0.293 | 0.806 | .85 | .924 | .358 |
|  | Depression | -.120 | .266 | -0.618 | 0.397 | -.046 | -.453 | .652 |
|  | SUD | -.053 | .308 | -0.578 | 0.596 | -.016 | -.172 | .864 |
|  | Bipolar | -.400 | .740 | -1.838 | 1.043 | -.050 | -.541 | .589 |
|  | Other | .444 | .375 | -0.281 | 1.179 | .133 | 1.182 | .239 |
|  | Gender | .242 | .237 | -0.212 | 0.689 | .097 | 1.018 | .311 |
|  | AgeD | .018 | .011 | -0.002 | 0.040 | .155 | 1.662 | .099 |

Supplement Table 3: Coefficients and statistics of independent variables of the multiple logistic regression model for prediction of µ n=114.

|  | | not standardised coefficients | | 95% Confidence intervalls of B | | Standardised coefficients | T | Sig. |
| --- | --- | --- | --- | --- | --- | --- | --- | --- |
|  |  | Regression-coefficient B | Std.-Error | Lower bound | Upper bound | β |  |  |
|  | (constant) | 372.658 | 65.492 | 242.955 | 502.361 |  | 5.690 | <0.001 |
|  | ADHD | 14.868 | 32.164 | -48.830 | 78.566 | .42 | .462 | .645 |
|  | Depression | -8.220 | 30.163 | -67.957 | 51.517 | -.027 | -.273 | .786 |
|  | SUD | -11.194 | 34.975 | -80.459 | 58.071 | -.030 | -.320 | .749 |
|  | Bipolar | -116.134 | 84.008 | -282.508 | 50.240 | -.126 | -1.382 | .169 |
|  | Other | 77.340 | 42.615 | -7.057 | 161.736 | .172 | 1.815 | .072 |
|  | Gender | 15.810 | 26.954 | -37.571 | 69.192 | .056 | .587 | .559 |
|  | AgeD | 1.738 | 1.221 | -0.680 | 4.156 | .132 | 1.423 | .157 |

Supplement Table 4: Coefficients and statistics of independent variables of the multiple logistic regression model for prediction of σ n=114.

|  | | not standardised coefficients | | 95% Confidence intervalls of B | | Standardised coefficients | T | Sig. |
| --- | --- | --- | --- | --- | --- | --- | --- | --- |
|  |  | Regression-coefficient B | Std.-Error | Lower bound | Upper bound | β |  |  |
|  | (constant) | 19.600 | 22.882 | -25.717 | 64.916 |  | .857 | .393 |
|  | ADHD | 14.084 | 11.238 | -8.172 | 36.339 | .110 | 1.253 | .213 |
|  | Depression | -.723 | 10.539 | -21.594 | 20.149 | -.007 | -.069 | .954 |
|  | SUD | 1.918 | 12.220 | -22.283 | 26.118 | .014 | .157 | .876 |
|  | Bipolar | -52.350 | 29.351 | -110.479 | 5.778 | -.157 | -1.784 | .077 |
|  | Other | 35.901 | 14.889 | 6.414 | 65.388 | .222 | 2.411 | **.017** |
|  | Gender | 14.465 | 9.417 | -4.186 | 33.116 | .141 | 1.536 | .127 |
|  | AgeD | .607 | .427 | -0.238 | 1.452 | .128 | 1.424 | .157 |

Supplement Table 5: Coefficients and statistics of independent variables of the multiple logistic regression model for prediction of τ n=114.

|  | | not standardised coefficients | | 95% Confidence intervalls of B | | Standardised coefficients | T | Sig. |
| --- | --- | --- | --- | --- | --- | --- | --- | --- |
|  |  | Regression-coefficient B | Std.-Error | Lower bound | Upper bound | β |  |  |
|  | (constant) | 168.126 | 26.34 | 116.171 | 220.082 |  | 6.409 | <0.001 |
|  | ADHD | 5.249 | 12.884 | -20.267 | 30.765 | .038 | .407 | .684 |
|  | Depression | -3.025 | 12.083 | -26.954 | 20.904 | -.026 | -.250 | .803 |
|  | SUD | 7.993 | 14.010 | -19.753 | 35.739 | -.054 | .571 | .569 |
|  | Bipolar | 43.117 | 33.652 | -23.528 | 109.762 | -.119 | 1.281 | .203 |
|  | Other | -1.729 | 17.071 | -35.537 | 32.078 | -.010 | -.101 | .919 |
|  | Gender | -.275 | 10.797 | -21.659 | 21.108 | .002 | -.026 | .980 |
|  | AgeD | -.247 | .489 | -1.215 | 0.722 | -.048 | -.505 | .615 |
